# The Ribosome Hypothesis: Decoding Mood Disorder Complexity

**DOI:** 10.3390/ijms25052815

**Published:** 2024-02-29

**Authors:** Vandana Sharma, Karthik Swaminathan, Rammohan Shukla

**Affiliations:** 1 Department of Zoology and Physiology, University of Wyoming, Laramie, WY 82071, USA; kswamina@uwyo.edu; 2 Department of Neurosciences, University of Wyoming, Laramie, WY 82071, USA

**Keywords:** ribosomes, ribosomal heterogeneity, stress, mood disorder

## Abstract

Several types of mood disorders lie along a continuum, with nebulous boundaries between them. Understanding the mechanisms that contribute to mood disorder complexity is critical for effective treatment. However, present treatments are largely centered around neurotransmission and receptor-based hypotheses, which, given the high instance of treatment resistance, fail to adequately explain the complexities of mood disorders. In this opinion piece, based on our recent results, we propose a ribosome hypothesis of mood disorders. We suggest that any hypothesis seeking to explain the diverse nature of mood disorders must incorporate infrastructure diversity that results in a wide range of effects. Ribosomes, with their mobility across neurites and complex composition, have the potential to become specialized during stress; thus, ribosome diversity and dysregulation are well suited to explaining mood disorder complexity. Here, we first establish a framework connecting ribosomes to the current state of knowledge associated with mood disorders. Then, we describe the potential mechanisms through which ribosomes could homeostatically regulate systems to manifest diverse mood disorder phenotypes and discuss approaches for substantiating the ribosome hypothesis. Investigating these mechanisms as therapeutic targets holds promise for transdiagnostic avenues targeting mood disorders.

## 1. Introduction

The global impact of mood disorders is significant, with major depressive disorder (MDD) alone affecting approximately 280 million people worldwide [1]. Mood disorders encompass a spectrum of depression subtypes [2], such as bipolar I depression, bipolar II depression, mixed depression, agitated depression, atypical depression, melancholic depression, recurrent brief depression, minor depressive disorder, seasonal depression, and dysthymic disorder, which exist along a continuum with nebulous boundaries. While these types are often treated as a single clinical entity in primary care [3], uncovering the mechanisms that differentiate them is crucial for the development of effective treatment strategies.

Several central nervous system components, including GABA, glutamate, monoaminergic neurotransmission, and the immune system, have been associated with depression and the target of medication strategies. However, the prevalence of MDD and treatment-resistant depression has continued to rise despite the use of these medications [4]. This persistent trend implies that these systems may not comprehensively elucidate the underlying biology of mood disorders. Understanding mood disorder complexity is crucial for the development of targeted treatment approaches that address specific depression subtypes.

In our recent comparative study, which examined the molecular similarities among in vitro and in vivo experimental models of chronic stress, recapitulating several aspects of depression, and postmortem subjects with mood disorders [5], we observed the significant dysregulation of ribosomal protein genes (RPGs) across all these paradigms. Based on this finding, we propose a ribosome hypothesis of mood-related disorders. The hypothesis suggests that RPG dysregulation during these disorders can alter the number and composition of ribosomes, potentially leading to stress-induced ribosome specialization. This specialization, particularly in neurites, can impact the synthesis of proteins that sculpt neuronal input and output, contributing to various forms of synaptic dysregulation associated with mood disorders.

In this opinion piece, we aim to further examine how the ribosome, with its diverse composition and regulatory mechanisms at different levels, fits into the existing anatomical and physiological frameworks of mood-related disorders and has the potential to offer explanations for depression subtype complexity. We discuss the development of a detailed model of potential mechanisms of ribosomal regulation in mood disorders and address the outstanding questions and associations that can establish the foundational aspects of the ribosome hypothesis of mood-related disorders.

## 2. The Role of Ribosomes within the Current Anatomical and Physiological Frameworks of Mood Disorders

Several broad frameworks have been proposed to describe the pathophysiology of mood disorders at different levels. In this section, we will examine the existing anatomical and physiological frameworks of mood disorders and present findings that position ribosomes and their infrastructure within these frameworks.

At the anatomical level, the diverse structure and branching pattern of dendrites play a significant role in shaping input integration, including excitatory and inhibitory inputs from different branches over time [6,7]. Previous studies have shown that abnormalities in dendritic input or regulation can cause microcircuitry remodeling, leading to cognitive changes associated with learning, memory, and attention deficits, which are hallmarks of mood disorders [8,9,10]. In our recent study [5], we discovered an inverse relationship between the expression of RPGs and their pseudogenes, with RPGs being downregulated and RP pseudogenes being upregulated, in three paradigms: (1) postmortem subjects with MDD and those who died by suicide; (2) mice exposed to chronic variable stress (CVS); and (3) glucocorticoid-stressed primary neurons. Recent neuronal compartment-specific transcriptomic studies have demonstrated the enrichment of RPGs in the dendrites and neuritic compartment of neurons compared to the soma [11,12,13,14,15]. Strikingly, across all three paradigms, the observed dysregulation of RPG families prominently correlated with the neurite and synaptic pathways, establishing a direct link between compartment-specific enrichment and functional impact. Additionally, confirming its functional association in each of the respective paradigms, the inverse relationship between RPGs and RP pseudogenes was (1) reversed in postmortem subjects in remission and attenuated in (2) CVS-exposed mice treated with ketamine, a rapid antidepressant, and (3) glucocorticoid-stressed primary neurons treated with RU486, a glucocorticoid receptor antagonist. While ribosomes are traditionally viewed as homogeneous entities, building on the existing ribosome filter hypothesis [16], we propose that the functional dysregulations resulting from stress-induced changes in RP expression may lead to altered ribosome composition and the formation of specialized ribosomes [17,18] that can differentially impact mRNA translation during these disorders. In neurites, the ribosome specialization may alter protein synthesis to influence synaptic input and output.

At the physiological level, acute and chronic environmental changes can induce various types of plasticity in neuronal networks, and these types of plasticity are thought to underlie some of the manifestations of mood disorders [19]. Hebbian plasticity, for example, involves changes in synaptic strength that align with the applied stimuli in a feedforward manner. Strong stimulation leads to long-term potentiation (LTP), while sustained low-frequency stimulation results in long-term depression (LTD) [20]. On the other hand, homeostatic plasticity (aka, homeostatic synaptic scaling) utilizes negative feedback to restore neuronal activity patterns to their initial set point by adjusting synaptic strengths in the opposite direction [21,22]. For instance, global silencing activity increases synaptic strength, while enhancing activity decreases efficacy. Homeostatic plasticity is crucial for maintaining optimal neural function by preserving relative synaptic strengths, which is particularly important for cognitive functions. Consequently, homeostatic plasticity holds promise as a potential target for treating cognitive impairments associated with mood-related disorders [23]. Notably, homeostatic plasticity relies on the synthesis of new proteins that regulate key physiological parameters and takes time to develop, ranging from hours to days [24,25,26]. At the structural level, homeostatic plasticity is a result of both neuron-wide and compartment-specific changes; importantly, the crucial regulators of synaptic scaling exhibit inverse associations in their expression in the soma and neurites. In this context, ribosomes, as contributors to protein synthesis, may play a significant role in the mechanisms underlying the neuron-wide and compartment-specific changes that are the foundation of homeostatic plasticity. Supporting this notion, our study demonstrated that the dysregulated expression of RPGs was positively correlated with soma-related pathways and negatively correlated with neurite-related pathways. Furthermore, the average half-life of RPGs (10 to 17 h) [27] falls within the duration required for homeostatic scaling, further supporting their potential involvement in mechanisms of homeostatic synaptic scaling. Thus, stress-induced ribosome dysregulation may be a key contributor to the homeostatic plasticity underlying mood disorders.

We posit that any comprehensive framework explaining the diversity in mood disorders must incorporate the infrastructure diversity that results in a wide range of effects. Ribosomes, characterized by their intricate composition, numerous phosphorylation sites, and multiple rRNA molecules, constitute a crucial component of the neuronal infrastructure, which offers a vast repertoire of regulatory capabilities that can provide insights on the subtle differentiations observed among various types of mood disorders. In the following section, we will delve into the ribosome hypothesis of mood-related disorders, building upon these anatomical and physiological associations.

## 3. The Ribosome Hypothesis: Mechanisms of Ribosomal Regulation in Mood-Related Disorders

**Figure 1:**
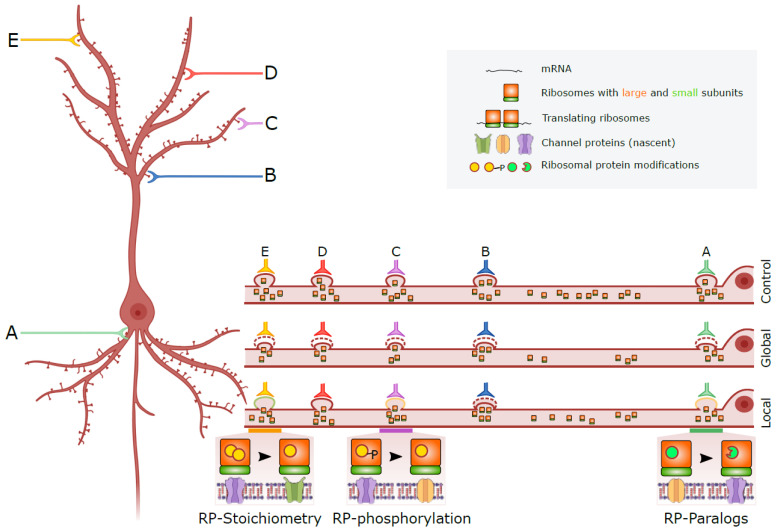
The potential mechanisms by which the downregulation of RPGs could impact synaptic inputs (A–E) are outlined as follows: 1. Global Effects (Location Non-Specific): RPG downregulation is likely to lead to a reduction in ribosome production. This, in turn, may diminish the synthesis of synaptic proteins, ultimately resulting in an overall decrease in synaptic weight. 2. Local Effects (Location Specific): In addition to the global impact, RPG downregulation may induce changes in ribosome composition within specific cellular locales. This can manifest as the removal, alteration, or substitution of a few RPs. Such alterations have the potential to give rise to specialized ribosomes, thereby influencing the translation of synaptic proteins in a compartment-specific manner. 3. Concomitant Reduction in Ribosome Production: It is noteworthy that the reduction in ribosome production, as observed due to RPG downregulation, may occur simultaneously with the generation of specialized ribosomes. In such instances, the decrease in synaptic weight can be observed both globally and locally.

Our hypothesis posits that there are two primary mechanisms by which a change in RP expression can sculpt the adaptability of neurons to changes in synaptic input (Figure 1): (1) through a global alteration in ribosome biosynthesis that influences synaptic protein expression and synaptic weight throughout the neuron and (2) through local alterations in ribosome composition, leading to ribosome specialization that affects synaptic function in a compartment-specific manner.

First, the downregulation of RPGs can result in reduced ribosome biosynthesis throughout the neuron, leading to a global decrease in translation and protein synthesis. A similar downregulation of RPGs is observed in single-celled microorganisms as a strategy to conserve energy and nutrients during stress [28,29]. In MDD and CVS, RPG downregulation may preserve the essential amino acids abundant in ribosomes [30,31], such as lysine and arginine, deficiencies of which have been linked to depression [32,33,34]. As the majority of RPGs are enriched in neurites, the homeostatic downregulation of RPGs reduces global synaptic protein synthesis, thereby contributing to homeostatic synaptic scaling.

Homeostatic scaling can also occur in a set of synapses on a particular dendritic branch or in a particular synapse [23]. Thus, the second mechanism by which RPs can regulate neuronal response is through modifying ribosome composition to form specialized ribosomes in a site-specific manner. At the transcript level, there are over 80 RPGs and more than 2000 RP pseudogenes. Ribosome biogenesis, reliant on equimolar levels of RPs, is sensitive to RPG gene dosage. The downregulation of RPGs during stress and depression suggests that it could disrupt the molar quantity (stoichiometry) among the core RPs composing the ribosomes, thus introducing heterogeneity. Additionally, since the number and type of RPGs downregulated differed in a phenotype-, species-, and sex-specific manner [5], the heterogeneity extends to diverse regulatory patterns across these variables. RP stoichiometry can also be influenced by the upregulation of the RP pseudogenes observed in our study. At the transcript level, RP pseudogenes can act as short interfering RNA (siRNA) to downregulate the expression of parent RPGs through an RNA interference-based mechanism or function as competitive endogenous RNA (ceRNA), acting as sponges for common microRNAs (miRNAs) to attenuate the downregulation of parent RPG expression [35,36]. In either case, RP pseudogenes can modify the expression of specific RPGs, leading to altered ribosome stoichiometry. Most pseudogenes are also known to code for a truncated form of a protein [37,38,39,40], which can serve as an RP paralog and substitute for the parent RP in the ribosome, further adding to ribosome heterogeneity.

Ribosomal heterogeneity is distinct from ribosomal specialization. Specialized ribosomes are a subset of heterogeneous ribosomes that selectively modulate translation for specific mRNAs. They possess the ability to selectively and site-specifically regulate translation control [41], influencing translation initiation, speed, fidelity, and selectivity (Figure 1, bottom). Notably, specialized ribosomes, along with their ability to move across neurites, can generate numerous permutations that contribute to the diverse way in which the synaptic input can be regulated throughout the dendritic arbor, potentially explaining the nuanced nature of the spectrum of depression and other mood disorders.

## 4. Exploring Key Questions and Associations in the Ribosome Hypothesis

The proposed ribosome hypothesis raises several important questions that require further investigation. First, since the hypothesis is based on transcriptomics data, a critical first step is to examine the gene expression program (i.e., gene to protein) of RPGs and RP pseudogenes, elucidating their role in the global and local regulation of ribosomes. At the transcriptional level, research needs to identify stress-induced transcription factors and how they regulate the RPG dose (i.e., mRNA levels), which tightly controls RP stoichiometry. At the post-transcriptional level, studies need to investigate the role of RPG mRNA splicing in regulating RPG diversity and explore the role of RP pseudogenes as miRNAs and ceRNAs that modulate RPG mRNA processing. To establish the existence of the altered ribosome stoichiometry proposed in our ribosome hypothesis, investigations must identify the absolute change in RPs within ribosomes and define the specific RPs undergoing altered stochiometric composition using quantitative proteomics approaches [42,43]. As mentioned above, RP pseudogenes are likely to play an important role in ribosome heterogeneity and dysregulation; however, they pose a particular challenge in proteomics studies, as their similarity to the parent RP make them difficult to distinguish. Thus, translatiomics-based approaches, known for mitigating this issue [37], will be needed to establish the role of RP pseudogenes in coding for RP paralogs and altering ribosome stoichiometry [44]. Together, these investigations are likely to demonstrate stress-induced changes in ribosomal proteins, resulting in an altered ribosome number and composition, providing support for our ribosome hypothesis.

Our current understanding of mood disorders includes an appreciation for the cell type-specific changes underlying circuit dysregulation. Thus, single-cell transcriptomics (scRNA-seq) and translatomics (scRibo-seq) approaches should be employed to discern the cell-type-specific gene expression program of RPGs and RP pseudogenes. Next, the most challenging aspect of substantiating the ribosome hypothesis lies in establishing the role of site-specific ribosomal specialization in explaining the nuanced nature of the spectrum of mood disorders. Investigating site-specific ribosomal specialization entails generating permutations that modify synaptic input throughout the dendritic arbor. Unfortunately, there is a lack of experiments that simultaneously modify ribosomal composition and map the synaptic input at different dendritic arbor locations. However, with a mathematical understanding of protein mobility across dendrites [45] and well-rationalized assumptions regarding changes in synaptic scaling along the depression spectrum, available detailed biophysical models can be employed to examine and evaluate this concept.

Finally, genome-wide association studies (GWASs) of depression [46] have identified genetic variations linked to neurite- and synapse-related pathways, which act in parallel to the pathways associated with the dysregulation of RPG infrastructure. This intriguing association calls for an examination of the correspondence between the genetic variations identified in GWAS and RPG infrastructure (i.e., RPGs and RP pseudogenes). Understanding the degree of correspondence between depression-related genetic variants and RPG infrastructure will provide insights into whether the observed synaptic changes associated with RPG infrastructure are driven by genetic mechanisms underlying depression risk or by pathophysiological mechanisms that influence symptom manifestations.

## 5. Conclusions

In conclusion, the ribosome hypothesis capitalizes on the evolutionarily conserved role of ribosomes and RPGs in stress [47], offering valuable insight into the nuances linked with various depression subtypes. Furthermore, the conserved nature of this dysregulation in human MDD and mouse models of stress provides a simple experimental system for further the investigation of the proposed mechanisms. The diversity of RPGs and their regulatory role at various levels are key aspects of this hypothesis and suggest that targeting RP expression and ribosomal specialization could inspire unexplored therapeutic strategies. Notably, ribosomes, typically associated with protein synthesis, are involved in synthesizing transmitter receptors, synaptic scaffold proteins, and other regulatory proteins. Therefore, our novel ribosome hypothesis encompasses and integrates other existing hypotheses of depression involving GABA [48], glutamate [49], immune [50], and monoaminergic [51] systems. As such, exploring the therapeutic options associated with ribosomal dysregulation holds promise for a broader transdiagnostic impact across depression subtypes.

## References

[1] World Health Organization (2023). Depressive Disorder (Depression). https://www.who.int/news-room/fact-sheets/detail/depression.

[2] Angst J., Sellar R., Merikangas K.R. (2000). Depressive spectrum diagnoses. Compr. Psychiatry.

[3] Benazzi F. (2006). Various forms of depression. Dialogues Clin. Neurosci..

[4] Howes O.D., Thase M.E., Pillinger T. (2021). Treatment resistance in psychiatry: State of the art and new directions. Mol. Psychiatry.

[5] Zhang X., Eladawi M.A., Ryan W.G., Fan X., Prevoznik S., Devale T., Ramnani B., Malathi K., Sibille E., Mccullumsmith R. (2023). Ribosomal dysregulation: A conserved pathophysiological mechanism in human depression and mouse chronic stress. PNAS Nexus.

[6] Nelson S., Greg S., Michael H. (2016). Principles of dendritic integration. Dendrites.

[7] Cuntz H., Borst A., Segev I. (2007). Optimization principles of dendritic structure. Theor. Biol. Med. Model..

[8] Forrest M.P., Parnell E., Penzes P. (2018). Dendritic structural plasticity and neuropsychiatric disease. Nat. Rev. Neurosci..

[9] Wang X.J., Krystal J.H. (2014). Computational psychiatry. Neuron.

[10] Smail M.A., Wu X., Henkel N.D., Eby H.M., Herman J.P., McCullumsmith R.E., Shukla R. (2021). Similarities and dissimilarities between psychiatric cluster disorders. Mol. Psychiatry.

[11] Cajigas I.J., Tushev G., Will T.J., Dieck S.T., Fuerst N., Schuman E.M. (2012). The local transcriptome in the synaptic neuropil revealed by deep sequencing and high-resolution imaging. Neuron.

[12] Gumy L.F., Yeo G.S., Tung Y.-C.L., Zivraj K.H., Willis D., Coppola G., Lam B.Y., Twiss J.L., Holt C.E., Fawcett J.W. (2010). Transcriptome analysis of embryonic and adult sensory axons reveals changes in mRNA repertoire localization. RNA.

[13] Poon M.M., Choi S.-H., Jamieson C.A.M., Geschwind D.H., Martin K.C. (2006). Identification of process-localized mRNAs from cultured rodent hippocampal neurons. J. Neurosci..

[14] Zhong J., Zhang T., Bloch L.M. (2006). Dendritic mRNAs encode diversified functionalities in hippocampal pyramidal neurons. BMC Neurosci..

[15] Perez J.D., Tom Dieck S., Alvarez-Castelao B., Tushev G., Chan I.C.W., Schuman E.M. (2021). Subcellular sequencing of single neurons reveals the dendritic transcriptome of GABAergic interneurons. eLife.

[16] Mauro V.P., Edelman G.M. (2002). The ribosome filter hypothesis. Proc. Natl. Acad. Sci. USA.

[17] Xue S., Barna M. (2012). Specialized ribosomes: A new frontier in gene regulation and organismal biology. Nat. Rev. Mol. Cell Biol..

[18] Slavov N., Semrau S., Airoldi E., Budnik B., van Oudenaarden A. (2015). Differential Stoichiometry among Core Ribosomal Proteins. Cell Rep..

[19] Shukla R., Newton D.F., Sumitomo A., Zare H., Mccullumsmith R., Lewis D.A., Tomoda T., Sibille E. (2022). Molecular characterization of depression trait and state. Mol. Psychiatry.

[20] Malenka R.C., Bear M.F. (2004). LTP and LTD: An embarrassment of riches. Neuron.

[21] Turrigiano G.G., Leslie K.R., Desai N.S., Rutherford L.C., Nelson S.B. (1998). Activity-dependent scaling of quantal amplitude in neocortical neurons. Nature.

[22] Turrigiano G.G., Nelson S.B. (2004). Homeostatic plasticity in the developing nervous system. Nat. Rev. Neurosci..

[23] Kavalali E.T., Monteggia L.M. (2020). Targeting Homeostatic Synaptic Plasticity for Treatment of Mood Disorders. Neuron.

[24] Turrigiano G. (2012). Homeostatic Synaptic Plasticity: Local and Global Mechanisms for Stabilizing Neuronal Function. Cold Spring Harb. Perspect. Biol..

[25] Fernandes D., Carvalho A.L. (2016). Mechanisms of homeostatic plasticity in the excitatory synapse. J. Neurochem..

[26] Dubes S., Favereaux A., Thoumine O., Letellier M. (2019). miRNA-Dependent Control of Homeostatic Plasticity in Neurons. Front. Cell. Neurosci..

[27] Sharova L.V., Sharov A.A., Nedorezov T., Piao Y., Shaik N., Ko M.S.H. (2009). Database for mRNA half-life of 19977 genes obtained by DNA microarray analysis of pluripotent and differentiating mouse embryonic stem cells. DNA Res..

[28] Pontes M.H., Yeom J., Groisman E.A. (2016). Reducing Ribosome Biosynthesis Promotes Translation during Low Mg 2+ Stress. Mol. Cell.

[29] Albert B., Kos-Braun I.C., Henras A.K., Dez C., Rueda M.P., Zhang X., Gadal O., Kos M., Shore D. (2019). A ribosome assembly stress response regulates transcription to maintain proteome homeostasis. eLife.

[30] An H., Harper J.W. (2020). Ribosome Abundance Control Via the Ubiquitin–Proteasome System and Autophagy. J. Mol. Biol..

[31] Riba A., Di Nanni N., Mittal N., Arhné E., Schmidt A., Zavolan M. (2019). Protein synthesis rates and ribosome occupancies reveal determinants of translation elongation rates. Proc. Natl. Acad. Sci. USA.

[32] Ali-Sisto T., Tolmunen T., Viinamäki H., Mäntyselkä P., Valkonen-Korhonen M., Koivumaa-Honkanen H., Honkalampi K., Ruusunen A., Nandania J., Velagapudi V. (2018). Global arginine bioavailability ratio is decreased in patients with major depressive disorder. J. Affect. Disord..

[33] Fan M., Gao X., Li L., Ren Z., Lui L.M.W., McIntyre R.S., Teopiz K.M., Deng P., Cao B. (2021). The Association Between Concentrations of Arginine, Ornithine, Citrulline and Major Depressive Disorder: A Meta-Analysis. Front. Psychiatry.

[34] Smriga M., Kameishi M., Uneyama H., Torii K. (2002). Dietary L-lysine deficiency increases stress-induced anxiety and fecal excretion in rats. J. Nutr..

[35] An Y., Furber K.L., Ji S. (2016). Pseudogenes regulate parental gene expression *via* ceRNA network. J. Cell. Mol. Med..

[36] Tam O.H., Aravin A.A., Stein P., Girard A., Murchison E.P., Cheloufi S., Hodges E., Anger M., Sachidanandam R., Schultz R.M. (2008). Pseudogene-derived small interfering RNAs regulate gene expression in mouse oocytes. Nature.

[37] Ji Z., Song R., Regev A., Struhl K. (2015). Many lncRNAs, 5′UTRs, and pseudogenes are translated and some are likely to express functional proteins. eLife.

[38] Zhang Z., Harrison P., Gerstein M. (2002). Identification and analysis of over 2000 ribosomal protein pseudogenes in the human genome. Genome Res..

[39] Vanin E.F. (1985). Processed pseudogenes: Characteristics and evolution. Annu. Rev. Genet..

[40] Mighell A.J., Smith N.R., Robinson P.A., Markham A.F. (2000). Vertebrate pseudogenes. FEBS Lett..

[41] Genuth N.R., Barna M. (2018). The Discovery of Ribosome Heterogeneity and Its Implications for Gene Regulation and Organismal Life. Mol. Cell.

[42] Emmott E., Jovanovic M., Slavov N. (2019). Approaches for Studying Ribosome Specialization. Trends Biochem. Sci..

[43] Petelski A.A., Slavov N. (2020). Analyzing Ribosome Remodeling in Health and Disease. Proteomics.

[44] Alkan F., Wilkins O.G., Hernández-Pérez S., Ramalho S., Silva J., Ule J., Faller W.J. (2022). Identifying ribosome heterogeneity using ribosome profiling. Nucleic Acids Res..

[45] Fonkeu Y., Kraynyukova N., Hafner A.-S., Kochen L., Sartori F., Schuman E.M., Tchumatchenko T. (2019). How mRNA Localization and Protein Synthesis Sites Influence Dendritic Protein Distribution and Dynamics. Neuron.

[46] Howard D.M., Adams M.J., Clarke T.-K., Hafferty J.D., Gibson J., Shirali M., Coleman J.R.I., Hagenaars S.P., Ward J., Wigmore E.M. (2019). Genome-wide meta-analysis of depression identifies 102 independent variants and highlights the importance of the prefrontal brain regions. Nat. Neurosci..

[47] Ghulam M.M., Catala M., Elela S.A. (2020). Differential expression of duplicated ribosomal protein genes modifies ribosome composition in response to stress. Nucleic Acids Res..

[48] Luscher B., Shen Q., Sahir N. (2011). The GABAergic deficit hypothesis of major depressive disorder. Mol. Psychiatry.

[49] Moriguchi S., Takamiya A., Noda Y., Horita N., Wada M., Tsugawa S., Plitman E., Sano Y., Tarumi R., ElSalhy M. (2019). Glutamatergic neurometabolite levels in major depressive disorder: A systematic review and meta-analysis of proton magnetic resonance spectroscopy studies. Mol. Psychiatry.

[50] Maes M. (1995). Evidence for an immune response in major depression: A review and hypothesis. Prog. Neuro-Psychopharmacol. Biol. Psychiatry.

[51] Hirschfeld R.M. (2000). History and evolution of the monoamine hypothesis of depression. J. Clin. Psychiatry.

